# The chromatin remodelling factor BRG1 is a novel binding partner of the tumor suppressor p16^INK4a^

**DOI:** 10.1186/1476-4598-8-4

**Published:** 2009-01-16

**Authors:** Therese M Becker, Sebastian Haferkamp, Menno K Dijkstra, Lyndee L Scurr, Monika Frausto, Eve Diefenbach, Richard A Scolyer, David N Reisman, Graham J Mann, Richard F Kefford, Helen Rizos

**Affiliations:** 1 Westmead Institute for Cancer Research, University of Sydney, Westmead Millennium Institute and Westmead Hospital, Westmead, NSW 2145, Australia; 2 Westmead Millennium Institute, University of Sydney Westmead, NSW 2145, Australia; 3 Sydney Melanoma Unit, University of Sydney NSW Australia; 4 Department of Medicine, University of Michigan, Ann Arbor Michigan 48109, USA

## Abstract

**Background:**

*CDKN2A/p16*^*INK4a *^is frequently altered in human cancers and it is the most important melanoma susceptibility gene identified to date. p16^INK4a ^inhibits pRb phosphorylation and induces cell cycle arrest, which is considered its main tumour suppressor function. Nevertheless, additional activities may contribute to the tumour suppressor role of p16^INK4a ^and could help explain its specific association with melanoma predisposition. To identify such functions we conducted a yeast-two-hybrid screen for novel p16^INK4a ^binding partners.

**Results:**

We now report that p16^INK4a ^interacts with the chromatin remodelling factor BRG1. We investigated the cooperative roles of p16^INK4a ^and BRG1 using a panel of cell lines and a melanoma cell model with inducible p16^INK4a ^expression and BRG1 silencing. We found evidence that BRG1 is not required for p16^INK4a^-induced cell cycle inhibition and propose that the p16^INK4a^-BRG1 complex regulates BRG1 chromatin remodelling activity. Importantly, we found frequent loss of BRG1 expression in primary and metastatic melanomas, implicating this novel p16^INK4a ^binding partner as an important tumour suppressor in melanoma.

**Conclusion:**

This data adds to the increasing evidence implicating the SWI/SNF chromatin remodelling complex in tumour development and the association of p16^INK4a ^with chromatin remodelling highlights potentially new functions that may be important in melanoma predisposition and chemoresistance.

## Background

The cyclin dependent kinase inhibitor p16^INK4a ^is frequently inactivated in human cancers and is a highly penetrant melanoma susceptibility gene; more than 50 germline mutations have been identified in high-risk melanoma-prone families [1]. The principal function of p16^INK4a ^is to inhibit cell cycle progression by preventing the cyclin dependent kinases CDK4 and CDK6 from phosphorylating the retinoblastoma protein, pRb. In the presence of p16^INK4a^, pRb remains hypophosphorylated and forms active pRb-E2F transcriptional repressor complexes that silence genes required for S-phase entry. Consequently, ectopic expression of p16^INK4a ^promotes pRb-dependent G1 cell cycle arrest and senescence. Moreover, functional p16^INK4a ^is commonly maintained in pRb-deficient tumors (reviewed by Sherr & Roberts [2]), and this underscores the dependency of p16^INK4a ^on the pRb pathway.

Hypophosphorylated pRb can repress gene transcription at least partly by remodelling chromatin structure through its interactions with proteins such as HDAC1, BRM and BRG1 [3-5]. As the catalytic core of the SWI/SNF chromatin remodelling complex, the interaction between BRG1 and pRb was proposed to recruit the complex to E2F responsive promoters and enhance pRb transcriptional repressor activity. [5] There is also evidence that BRG1 acts upstream of pRb by repressing cyclin D1 expression [6] and upregulating the expression of the CDK inhibitors p21^Waf1^, p15^INK4b ^and p16^INK4a ^[7-9] to maintain pRb in an active, hypophosphorylated state. Not surprisingly, BRG1 may function as a tumor suppressor; BRG1 hemizygous mice are susceptible to tumors [10], while complete loss of BRG1 potentiates lung cancer development [11] and BRG1 is silenced or mutated in human tumor cell lines derived from breast, ovarian, lung, brain and colon cancers [4,12]. BRG1 is also lost in established neuroendocrine carcinomas and adenocarcinomas of the cervix [13], and the loss of BRG1 expression in lung cancer is associated with a poor prognosis [14,15].

In this study, it is identified for the first time that BRG1 specifically associates with p16^INK4a ^*in vivo*, and that both proteins are frequently lost in human melanomas. Although both BRG1 and p16^INK4a ^regulate pRb activity we found no evidence that p16^INK4a ^and BRG1 co-operate in cell cycle regulation. Targeted silencing of BRG1 did not diminish pRb-dependent p16^INK4a ^activities; p16^INK4a ^retained potent cell cycle inhibitory activity and induced senescence in the presence and absence of BRG1. Contrary to previous reports, that BRG1-deficient cells are relatively resistant to p16^INK4a^-induced cell cycle arrest [16], we show that pRb activity is BRG1-independent and thus, BRG1 does not influence p16^INK4a^-mediated cell cycle inhibition. Together with the frequent loss in primary melanomas the novel BRG1 interaction with the melanoma associated tumor suppressor p16^INK4a ^implies an important role for BRG1 in melanoma.

## Results

### BRG1 binds p16^INK4a^

**Figure 1:**
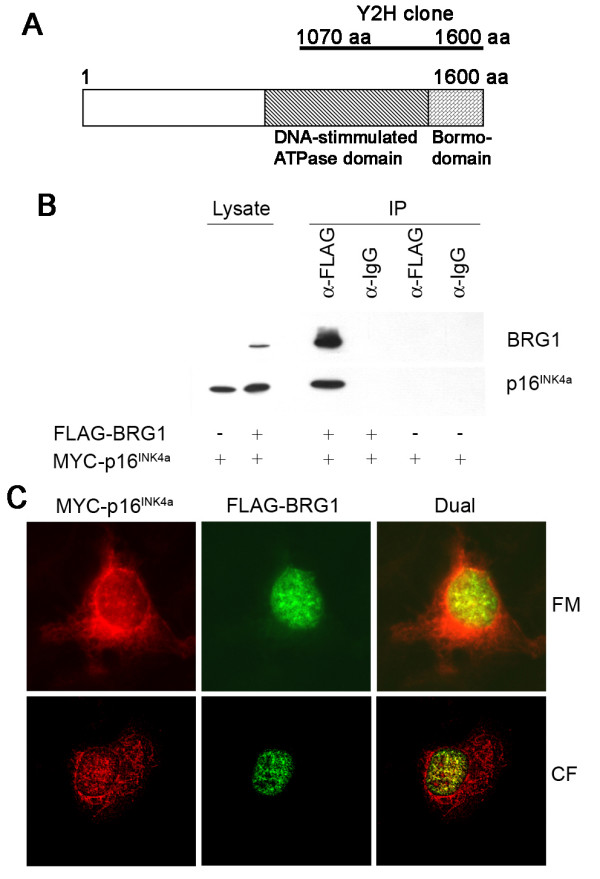
**Identification of BRG1 as p16^INK4a ^binding partner**. **A **Schematic illustration of BRG1 highlighting the domains isolated in the yeast 2-hybrid screen (Y2H clone) **B **U2OS cells were transfected with MYC-p16^INK4a ^and FLAG-BRG1 or control vector and immunoprecipitations were performed with a mouse-anti-FLAG antibody or a matched mouse IgG as indicated. BRG1 and p16^INK4a ^were detected on immunoblots with anti-FLAG and anti-MYC antibodies. **C **Fluorescent microscopy images (FM) and confocal microscopy images (CF) of SW-13 cells grown on cover slips and transfected with MYC-p16^INK4a ^and FLAG-BRG1 and probed with anti-FLAG and anti-MYC antibodies.

From a yeast two-hybrid screen using full-length human p16^INK4a ^as bait, we isolated the C-terminal 530 amino acids of the chromatin remodelling factor BRG1 as a potential binding partner (Figure 1A). This segment of BRG1 incorporates the ATPase domain, which facilitates ATP hydrolysis, and the bromodomain, which enables binding to acetylated histones [17]. To confirm that full-length BRG1 also binds p16^INK4a ^in human cells, both proteins were co-expressed transiently in U2OS osteosarcoma cells and MYC-tagged p16^INK4a ^was specifically co-purified with FLAG-tagged BRG1 in immunoprecipitation assays using a FLAG-specific antibody (Figure 1B). Further, when both proteins were co-expressed in the SW-13 adrenocortical carcinoma cell line, they co-localized in the nucleus in distinct nuclear speckles (Figure 1C).

**Figure 2:**
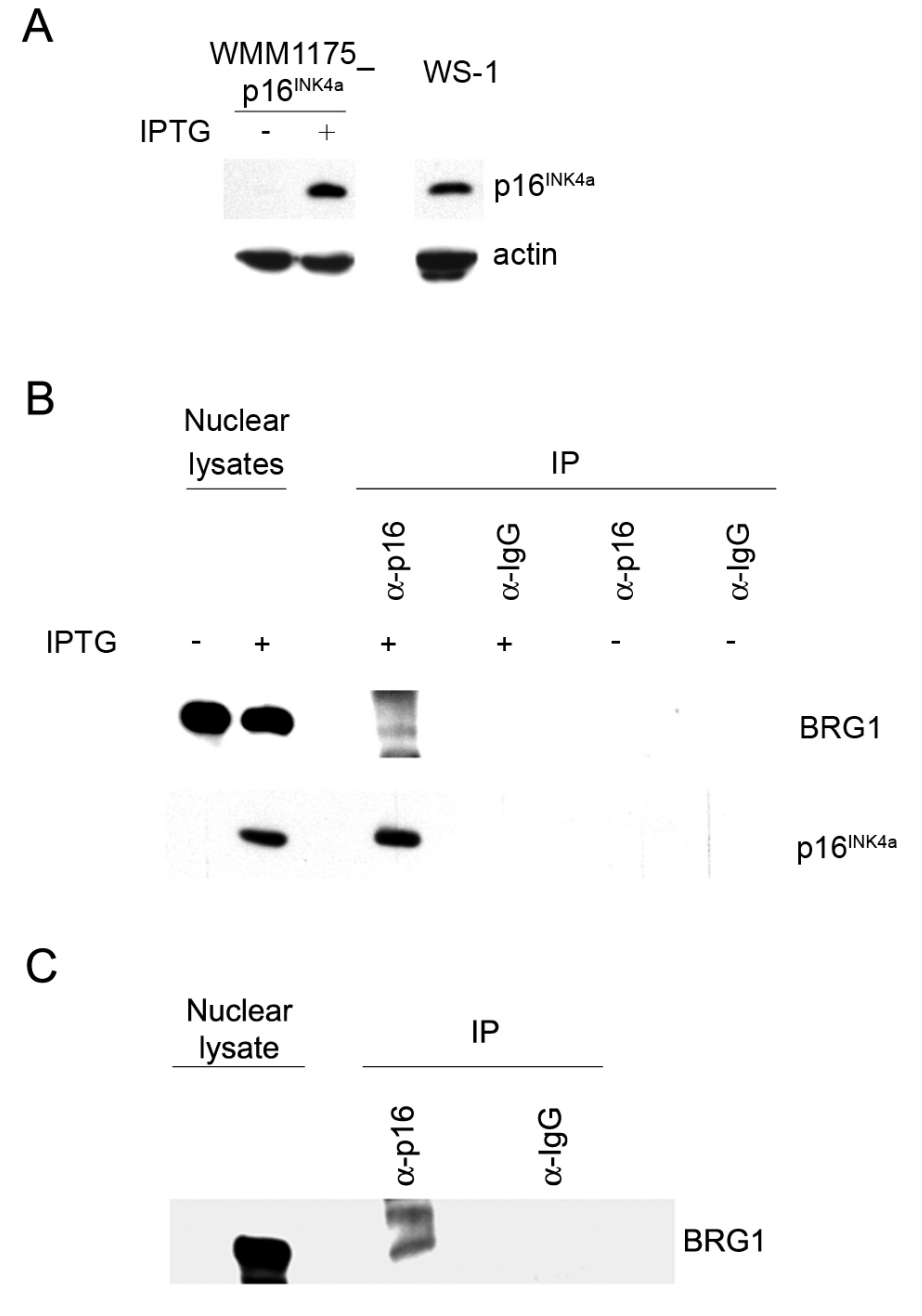
**BRG1 binds p16^INK4a ^in melanoma cells and normal fibroblasts**. **A **50 μg of total cell lysates derived from uninduced (-) and induced (+) WMM1175_p16^INK4a ^cells and WS-1 fibroblasts (passage 20) were separated using a 15% SDS-PAGE gel. Immunoblots were probed for p16^INK4a ^and β-actin as indicated. **B **WMM1175_p16^INK4a ^cells were induced to express p16^INK4a ^with 4 mM IPTG or mock treated for 72 hours. Immunoprecipitations were performed using a mouse anti-p16^INK4a ^antibody or a matched mouse IgG from nuclear cell lysate, as indicated. Immunoblots were probed for endogenous BRG1 and induced p16^INK4a ^using a mouse anti-BRG1 and rabbit anti-p16^INK4a^, respectively. **C **Endogenous BRG1 was co-immunoprecipitated with p16^INK4a ^from WS-1 normal dermal human fibroblasts grown to passage 20 as detailed above.

To verify that endogenous BRG1 also interacts with p16^INK4a^, we initially utilized the WMM1175_p16^INK4a ^inducible melanoma cell model, which we have previously described [18]. p16^INK4a ^expression was induced with IPTG to reach physiologically relevant levels comparable to those seen in the WS-1 normal human dermal fibroblasts at passage 20 (Figure 2A). Using a p16^INK4a^-specific antibody we isolated BRG1 from nuclear WMM1175_p16^INK4a ^lysates (Figure 2B). Importantly, the interaction between BRG1 and p16^INK4a ^was also confirmed in WS-1 normal human dermal fibroblasts at passage 20, using a p16^INK4a^-specific antibody (Figure 2C).

### pRb pathway in human cell lines

**Figure 3:**
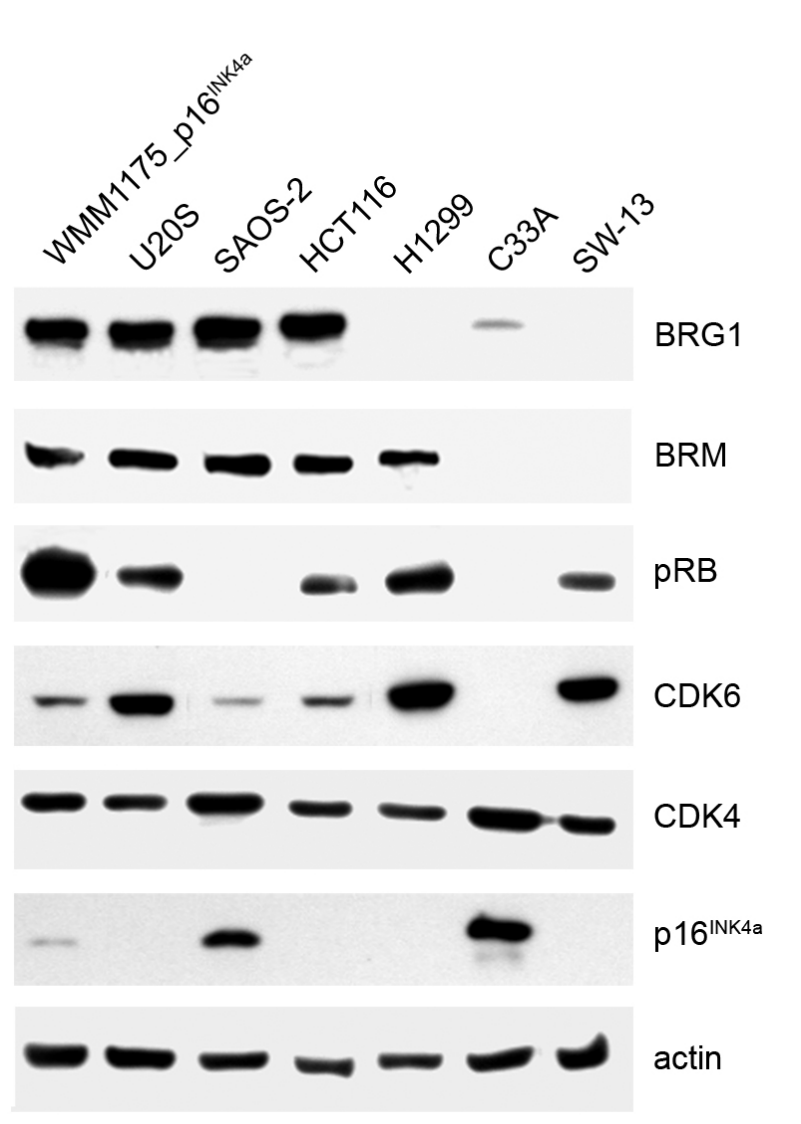
**pRb pathway proteins in cell lines**. Expression of BRG1 and BRM was analyzed using 50 μg of nuclear cell lysates. All other proteins were analyzed from 50 μg of total cell lysates.

To establish the role of BRG1 on p16^INK4a ^function we selected six cancer cell lines, varying in their p16^INK4a^, pRb and BRG1 status [12,16]. As shown in Figure 3 and Table 1, p16^INK4a ^expression was inversely related to pRb expression and only detected in the pRb-negative SAOS-2 osteosarcoma and C33A cervical cancer cells. All other cell lines had detectable pRb and no p16^INK4a^. (Note, there is a slight leakage of the ectopically introduced p16^INK4a ^in the p16-inducible WMM1175_p16^INK4a ^cells without IPTG upon long exposure.) The BRG1 homologue, BRM was expressed in all but the C33A cells and SW-13 adrenocortical carcinoma cells. Importantly, SW-13 and C33A cells were also negative or extremely low for BRG1 expression levels. The H1299 lung cancer cells were deficient for BRG1 expression, and all remaining cell lines had detectable levels of BRG1. It is also worth noting that the HCT116 cells carry only a mutated, functionally impaired BRG1 allele (BRG1^Leu1163Pro^) [12]. CDK4 was expressed strongly in all cell lines, while its homologue, CDK6 was either absent or poorly expressed in the pRb negative SAOS-2 and C33A cells and present in the remaining cells.

**Table 1:** pRb pathway proteins in cell lines

	**WMM1175-wtp16**	**U20S**	**Saos-2**	**HCT116**	**H1299**	**C33A**	**SW13**
**BRG1**	+	+	+	mutant	-	low	-

**BRM**	+	+	+	+	+	-	-

**pRb**	+	+	-	+	+	-	+

**CDK6**	low	+	low	low	+	-	+

**CDK4**	+	+	+	+	+	+	+

**p16**	**(-)**	-	+	-	-	+	-

Expression of the indicated proteins is summarized with + or -. Mutant status of BRG1 in HCT-116 cells has been reported [12].

### p16^INK4a ^requires pRB to induce cell cycle arrest

**Figure 4:**
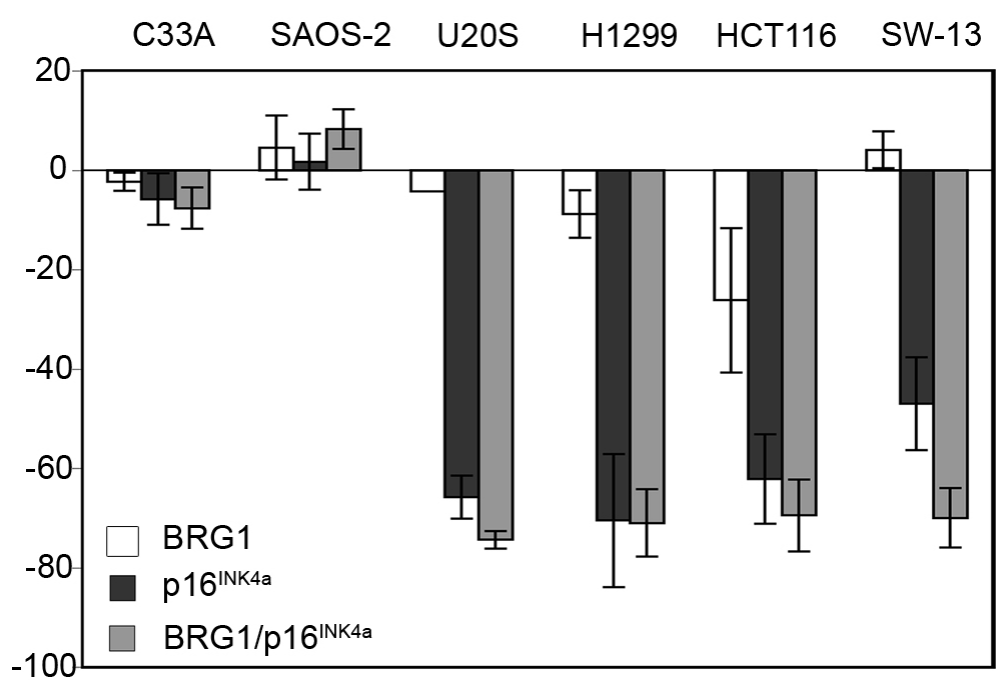
**BRG1 and p16^INK4a ^in cell cycle regulation**. Indicated cell lines were transfected with MYC-p16^INK4a^, FLAG-BRG1 and/or a control vector plus GFP-spectrin. Cells were fixed with 70% ethanol 48 hours post transfection and cellular DNA was stained with propidium iodide. Percent S-phase change of GFP-spectrin positive cells was calculated (percent S-phase vector control - percent S-phase sample) × 100/percent S-phase vector control.

To define the impact of BRG1 on p16^INK4a ^function we transiently expressed either BRG1, p16^INK4a ^or both proteins in this panel of six cell lines. The short-term expression of BRG1 alone had no effect on the cell cycle distribution of the cell lines tested. As expected, neither p16^INK4a ^alone nor p16^INK4a ^in combination with BRG1 promoted cell cycle arrest in cells deficient for pRb (SAOS-2 and C33A). In contrast, introduction of p16^INK4a ^induced potent cell cycle arrest in all cell lines expressing pRb (U2OS, H1299, HCT116, SW-13) even when the cells lacked BRG1 (H1299) or carried a reported mutant form of BRG1 (HCT116) [12]. Further, co-expression of BRG1 did not significantly enhance the p16^INK4a ^induced cell cycle arrest in the U20S, H1299 or HCT116 cells. Importantly even in SW13 cells, which lack both BRG1 and its homologue BRM, p16^INK4a ^expression alone induced a significant cell cycle arrest and this was enhanced to some extent by over-expressing BRG1 (Figure 4). These data confirm that p16^INK4a^-induced cell cycle arrest requires intact pRb, but not BRG1.

### p16^INK4a ^does not require BRG1 to promote cell cycle arrest or induce cell senescence

**Figure 5:**
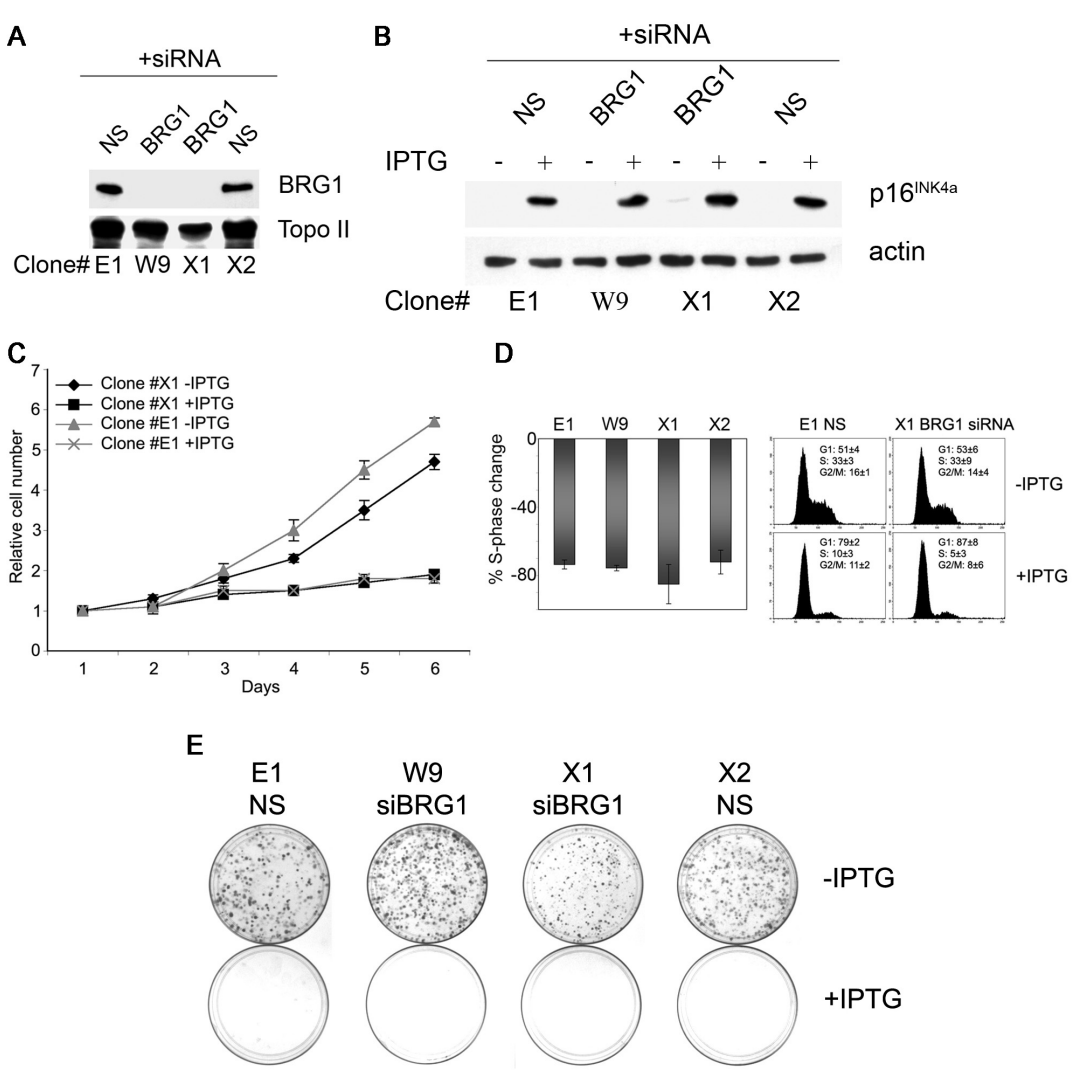
**BRG1 does not alter p16^INK4a ^cell cycle regulation**. **A **50 μg of nuclear lysates from WMM1175_p16^INK4a ^clones with stably integrated siRNA targeting BRG1 or a non-specific (NS) control siRNA were probed for BRG1 and topoisomerase II (Topo II) as a loading control. **B **50 μg of total cell lysates extracted from WMM1175_p16^INK4a ^cells stably expressing either a BRG1-specific siRNA or a non-specific (NS) siRNA molecule, as indicated, were treated with PBS (-) or IPTG (+) for 24 h and probed for p16^INK4a ^and β-actin. **C **Cell proliferation was determined by MTS assay. **D **A proportion of the IPTG/mock treated cells were analyzed for changes in cell cycle distribution. Percent S-phase change was calculated (percent S-phase mock treated cells – percent S-phase IPTG treated cells) × 100/percent S-phase mock treated cells. **E **The same clones were seeded at low density (10^3 ^cells/7.5 cm plate) and p16^INK4a ^expression was induced with 4 mM IPTG or cells mock treated and colony forming ability was assayed after 14 days.

To thoroughly evaluate any functional interaction between p16^INK4a ^and BRG1, we stably silenced BRG1 in the inducible WMM1175_p16^INK4a ^cell model. These cells were transfected with a silencing molecule specifically targeting BRG1 or a non-specific (NS) silencing molecule directed to the luciferase transcript. Two BRG1-silenced clones, WMM1175_p16^INK4a^_siBRG1 W9 and X1, with > 95% reduction in BRG1 accumulation and two control clones WMM1175_p16^INK4a^_sicontrol E1 and X2, with unaltered BRG1 expression, were selected for analysis. All clones remained inducible for p16^INK4a ^expression (Figure 5A, B).

Silencing of BRG1 had no significant impact on the proliferation rate or cell cycle distribution of the WMM1175_p16^INK4a ^cell line. In the absence of BRG1, p16^INK4a ^retained the ability to inhibit the proliferation of the WMM1175 cells (Figure 5C), and this was associated with arrest in the G1-phase of the cell cycle with a concomitant S-phase inhibition (Figure 5D) that was maintained over the five-day induction period (data not shown). Moreover, the silencing of BRG1 had no impact on the ability of p16^INK4a ^to totally prevent outgrowth of colonies upon low seeding density (Figure 5E).

**Figure 6:**
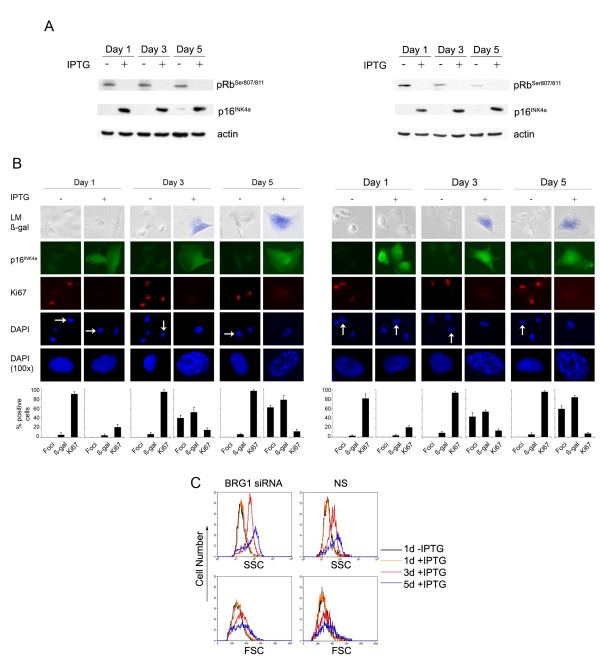
**BRG1 does not alter p16^INK4a ^driven senescence**. WMM1175_p16^INK4a ^cells, BRG1 silenced (clone X1, left panel) or NS (clone E1, right panel), were exposed to 4 mM IPTG over a five-day period and analyzed by FACS analysis, Western blot and imunocytostaining: **A **50 μg of total cell lysate were immunoblotted and probed for p16^INK4a^, phospho-pRb (pRbSer^807/811^) and as a loading control β-actin. **B **The accumulation of p16^INK4a^, the cell proliferation marker Ki67, chromatin condensation (DAPI) and the appearance of SA-β-gal was analyzed by immunocytostaining in WMM1175_p16^INK4a^. Enlarged images of cells (indicated with arrows) show DAPI-stained chromatin foci. Histograms correspond to the average ± s.d of at least two independent induction experiments from a total of at least 500 cells. LM, light microscopy. **C **FACS analysis by Forward Scatter (FSC) and Side Scatter (SSC) of clones demonstrate the senescence associated increase of cell size (FSC) and granularity (SSC) upon p16^INK4a ^induction.

BRG1 has been reported to induce senescence in SW-13 cells and in mesenchymal stem cells [7,19] and the role of p16^INK4a ^in initiating and maintaining senescence is widely acknowledged (reviewed by Huschtscha & Reddel [20]). We investigated the role of BRG1 in p16^INK4a^-induced senescence. The long term induction of p16^INK4a ^in WMM1175_p16^INK4a ^cells was not influenced by the BRG1 status, caused pRb hypophosphorylation (Figure 6A) and induced senescence-like features in the WMM1175 cells as reported previously [18,21], (Figure 6B). These features included increased cell size and granularity, positive senescence-associated β-galactosidase activity and the appearance of senescence-associated heterochromatin foci. Formation of foci coincides with the recruitment of pRb to E2F-responsive promoters and is associated with the stable repression of E2F-target genes [22]. This important marker of pRb activity was not affected by BRG1 silencing. Similarly, BRG1 silencing did not alter the build up of SA-β-galactosidase induced by p16^INK4a ^(Figure 6B) or p16^INK4a ^induced changes in cell size and granularity in the WMM1175 cells (Figure 6C), the latter corresponds to senescence associated vacuolisation. This data confirms that cell cycle regulation and induction of cell senescence by p16^INK4a ^does not require BRG1.

### BRG1 is lost in melanoma

**Figure 7:**
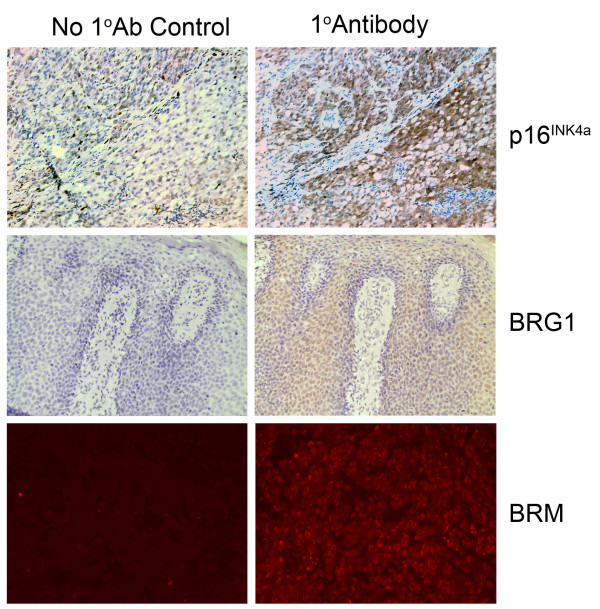
**Immunohistochemistry of melanomas for BRM, p16^INK4a ^and BRG1**. Melanoma samples were stained for p16^INK4a ^and BRG1 with immunohistochemistry using DAB. BRM was stained using red fluorescence. Positive staining examples are presented in the right panel with no primary antibody control from the corresponding region in the left panel.

To evaluate the role of BRG1 in melanomas, we examined immunohistochemically stained paraffin sections from archival paraffin-embedded tissue blocks of a series of primary and metastatic melanomas for expression of the chromatin remodelling factor BRG1 and p16^INK4a ^(Figure 7). As presented in Table 2, BRG1 expression was undetectable in 26/38 of melanomas (68%), whereas its homologue, BRM, was detected in 40/50 (80%) of melanoma specimens. As expected, p16^INK4a ^was only detected in a small proportion 20% (9/45) of these primary and metastatic melanomas. Of 21 tumor samples with expression data for p16^IN4a ^and BRG1 18 (86%) had lost at least one of these proteins, predominantly p16^INK4a^, and among these were 10 tumors (48%) negative for both, while 3 samples 14% had retained expression of both p16^INK4a ^and BRG1. BRM and BRG1 showed consistent nuclear localisation in all samples, while p16^INK4a ^was found to localise to the nucleus and cytoplasm. The proportion of BRG1 expression was slightly higher in the metastatic melanomas than in the primary melanomas, but, this did not reach significance using a Mann-Whitney Wilcoxon test. BRG1 and p16^INK4a ^were readily detectable in cultured, normal, primary human melanocytes (data not shown) and therefore our data imply that BRG1-loss has an important role in melanoma development.

**Table 2:** BRG1 is frequently lost in melanomas

	**BRM**	**p16^INK4a^**	**BRG1**
**Primary**	83% (19/23)	21% (5/24)	28% (5/18)

**Metastatic**	78% (21/27)	19% (4/21)	35% (7/20)

**Total**	80% (40/50)	20% (9/45)	32% (12/38)

Immunohistological detection of BRG1, p16^INK4a ^and BRM in primary and metastatic melanomas showing the proportion of samples with detectable positive staining.

## Discussion

The p16^INK4a ^tumor suppressor has a critical influence on melanoma tumorigenesis. We have now shown that the chromatin remodelling factor BRG1 is a novel binding partner of p16^INK4a^and confirm this interaction *in vivo*. More importantly, we show that loss of BRG1 occurs frequently in primary and metastatic melanomas and propose that BRG1 may play an important role as a tumor suppressor in this cancer.

We have also shown that p16^INK4a ^requires pRb, but not BRG1 to promote cell cycle arrest. This differs from several previous findings in the literature but agrees with others: It has been suggested that the pRb-BRG1 interaction is required for the pRb repression of E2F-target genes such as cyclin E and cyclin A, and thereby cell cycle arrest. According to this hypothesis, cells lacking BRG1 would harbor only inactive pRb, thus conferring resistance to p16^INK4a ^induced growth arrest [5,16]. These findings differ from those of Bultman et al. [23] who did not observe a functional interaction between pRB and BRG1 in their murine models and Kang et al. [7], who showed that the BRG1-pRB interaction was not required for BRG1 induced cell cycle arrest in SW-13 cells. In contrast to our work, Kang et al. [7] used long-term BRG1 expression, which caused growth arrest in SW-13 cells, and showed that BRG1 bound the p21Waf1 promoter and upregulated its expression 3–7 days after BRG1 expression. This was sufficient to induce cell cycle arrest and senescence independent of the BRG1 ability to complex with pRb. In this study we have clearly demonstrated that p16^INK4a ^requires pRb, but not BRG1, to promote cell cycle arrest. Our data is mainly based on the thorough analysis of a well-defined melanoma cell model, with inducible physiological relevant expression levels of p16^INK4a ^and the use of highly specific BRG1-silencing molecules. In this model, p16^INK4a ^induction promotes rapid G1-cell cycle arrest followed by cellular senescence, and these functions were not affected by silencing of BRG1.

Chromatin changes, which involve chromatin remodelling, are an important step in p16^INK4^/pRb dependent senescence [24]. It was recently shown that the BRG1 homologue, BRM, forms an initiating component of heterochromatin complexes during the senescence of melanocytes [25]. BRG1 has also been implicated in senescence of melanocytes, as it has been identified in the SWI/SNF complex facilitating transcription in response to IGFBP7, the latter itself being an important player in oncogenic BRAF-induced senescence [26]. However, our data show that p16^INK4a ^is able to promote senescence in WMM1175 melanoma cells in the absence of BRG1 indicating that the p16^INK4a^/pRb senescence pathway does not require BRG1.

As the catalytic component of the SWI/SNF chromatin remodelling complex, BRG1 facilitates unwinding of DNA helices bound to and wrapped around histone structures. The SWI/SNF chromatin remodelling complex can be recruited by specific DNA binding molecules such as transcriptional activators or repressors and directed to specific DNA targets. For instance, BRG1 promotes p53 dependent transcription by interacting with this tumor suppressor [27,28], while it functions as a co-repressor of E2F dependent transcription by associating with the E2F transcriptional repressor pRb [5]. Furthermore, BRG1 has recently been reported to promote transcriptional activity of the melanocyte specific transcription factor MITF-M [29]. MITF-M plays an important role in melanocyte proliferation and survival (reviewed by Goding) [30] and activates the expression of p16^INK4a ^[31]. It is possible that the p16^INK4a ^interaction with BRG1 modulates any one or more of these functions. For example it is tempting to speculate that p16^INK4a ^influences MITF-M transcriptional activity via its association with BRG1. This would create an important feedback loop between MITF-M and p16^INK4a^. We are currently investigating the impact of p16^INK4a ^on these BRG1 specific chromatin remodelling functions.

Regardless of the function of the BRG1-p16^INK4a ^complex, it is evident that BRG1 expression can be lost relatively early in melanoma development, with a significant proportion (> 70%) of primary melanomas showing no detectable BRG1 expression, while BRM expression was usually maintained in these tumors (< 20% loss). Overall, the rate of BRG1 loss was high in melanomas and comparable to that of p16^INK4a ^[32], which suggests that selection against BRG1 expression arises relatively early in melanoma genesis. The fact that, additionally to the frequent loss of either tumor suppressor, a high proportion of melanomas show loss of both proteins correlates with our data showing BRG1-independence of the p16^INK4a ^cell cycle regulatory functions and this suggests BRG1 independent and dependent functions of p16^INK4a^. BRG1 is proposed to be an important modulator of chromatin in melanocytic cells. In particular, BRG1 promotes transcriptional activity of the melanocyte specific transcription factor MITF-M [29], reduction of BRG1 expression in zebrafish embryos leads to a reduction in neural crest derived cells including melanocytes [33] and thirdly we found BRG1 expression in normal, primary human melanocytes. Therefore we propose that BRG1 is a vital melanoma associated tumour suppressor, which is frequently lost in the initial stages of the disease.

The identification of BRG1 as a potential tumor suppressor in melanoma adds to the increasing evidence implicating the SWI/SNF chromatin remodelling complex in tumor development. BRG1 mutations have been identified in small cell lung carcinomas [34] and loss of BRG1 expression or mislocalisation of BRG1 to the cytoplasm has been associated with poor prognosis in this malignancy [14,15]. Another study showed that 71% of neuroendocrine carcinomas of the cervix had lost BRG1 expression [13] and BRG1 has been implicated in breast cancer through its role in estrogen receptor dependent transcription [35], its interaction with the breast cancer susceptibility gene BRCA1 [27] and because BRG1 haploinsufficient mice are prone to mammary tumors [23]. Furthermore, BRG1 is often lost or mutated in various tumor cell lines including cells derived from pancreatic, ovarian, lung, brain and colon cancer [12]. In primary melanoma, the chromosomal region of BRG1 (19p13.2) is not deleted at high frequency [36], nevertheless, translocations in this chromosomal region have been associated with the disease in three cases [37].

## Conclusion

We have identified BRG1 as a novel binding partner of the tumor suppressor p16^INK4a ^and confirmed this interaction in normal cells. Together with our immunohistologic data confirming frequent BRG1 loss in primary melanomas, this implicates BRG1 as an important tumor suppressor in melanoma.

## Methods

### Yeast two-hybrid screen

The Matchmaker2 Gal4 yeast two-hybrid system (Clontech, Mountain View, CA, USA) was used to screen for p16^INK4a ^binding partners in the Y190 yeast strain with p16^INK4a ^cloned into the pAS2 vector in frame to the Gal4 binding domain and a human brain cDNA library cloned into the pACT2 vector in frame with the Gal4 transactivation domain (Clontech, Mountain View, CA, USA) according to the manufacturers instructions.

### Cell culture

U2OS, SAOS-2 (osteosarcoma), HCT116 (colon cancer), NCI-H1299 (lung cancer, are referred to as H1299 throughout this manuscript), C33A (cervical cancer), SW-13 (adrenocarcinoma), WS-1 (normal human fibroblasts) and WMM1175_wtp16 (melanoma) cells were grown in DMEM media with 10% foetal bovine serum and in case of WMM1175_wtp16 cells this was supplemented with 250 μg/ml Hygromycin and 500 μg/ml geneticin (Invitrogen, Carlsbad, CA, USA). Transfections were performed with FuGene (Roche, Mannheim, Germany).

### Stable BRG1 silenced p16^INK4a ^inducible WMM1175 clones

5 × 10^5 ^WMM1175_wtp16 cells were transfected with 4 μg of a BRG1 targeting siRNA (5'gatccGCATGCACCAGATGCACAAgttcaagagaCTTGTGCATCTGGTGCATGttttttggaaa3') cloned into the pSilencerPuro vector (Ambion, Austin, Texas, USA) or a control siRNA, targeting the luciferase gene, in the same vector supplied by Ambion. After selection with puromycin (2 μg/ml media) clones appeared after 20 days and were expanded, maintained with DMEM media including hygromycin, geneticin and puromycin and tested for BRG1 silencing and p16^INK4a ^inducibility.

### Antibodies

Mouse anti-β-actin (AC-74, Sigma, Castle Hill, NSW, Australia), mouse anti-Flag (M2, Sigma, Castle Hill, NSW, Australia), rabbit anti p16^INK4a ^antibody (Western and immunohistochemistry, N-20, SantaCruz, Santa Cruz, CA, USA), mouse anti-p16^INK4a ^antibody (immunoprecipitation, 2B4D11, Zymed Laboratories, San Francisco, CA, USA), mouse anti-BRG1 antibody (Western, G7, SantaCruz, Santa Cruz, CA, USA), rabbit anti-BRG1 antibody (immunohistochemistry, H-88, Santa Cruz, Santa Cruz, CA, USA), rabbit anti-MYC (A14, SantaCruz, Santa Cruz, CA, USA), Ki67 (MIB-1, Dako, Glostrup, Denmark), goat anti-BRM (Western, N-19, Santa Cruz, Santa Cruz, CA, USA), rabbit anti-BRM (immunohistochemistry, [38]), mouse anti-CDK4 (C8218, Sigma, Castle Hill, NSW, Australia), mouse anti-CDK6 (MS-451-P0, Neomarker, Union City, CA, USA), rabbit anti-phosphorylated pRb (Ser807/811, Cell Signalling, Boston, MA, USA), mouse anti-pRb (G3-245, BD Pharmingen, Franklin Lakes, NJ, USA), mouse anti-topoisomerase II (Ab1, Oncogene, San Diego, CA, USA),

### Immunoprecipitation

24 hours post seeding U2OS cells (2 × 10^6^), they were transfected with 7 μg pCMV-Myc5b-p16 and either 10.5 μg pcDNA3-BRG1-Flag [39] or 10.5 μg pCMV-Flag5b vector (Promega, Madison, Wisconsin, USA). Cells were harvested 24 hours post transfection, lysed in IP-buffer (50 mM Tris pH7.4, 150 mM NaCl, 1 mM EDTA, 1% NP-40, 0.25% sodium deoxycholate, protease inhibitors (Complete tablets, Roche, Mannheim, Germany)) and immunoprecipitation was performed with mouse-anti-Flag antibody or a matched mouse IgG coupled to tosyl-activated Dynal beads (Dynal Biotech, Oslo, Norway) following the manufacturers instructions. Proteins were separated on a 5–15% gradient SDS-PAGE gel, transferred to PVDF membranes (Millipore, Billerica, MA, USA) and probed for FLAG-BRG1 and MYC-p16^INK4a ^with the mouse-anti-FLAG antibody or a rabbit anti-p16^INK4a ^antibody.

For immunoprecipitations of endogenous BRG1 WMM1175_wtp16 cells were induced to express p16^INK4a ^with 4 mM IPTG or mock treated for 72 hours; alternatively passage 20 WS-1 human dermal fibroblasts were used. Nuclear pellets were produced using low salt buffer (10 mM HEPES pH 7.9, 10 mM KCl, 0.1 mM EDTA, 0.1 mM EGTA, 1 mM DTT) and lyzed in IP-buffer with protease inhibitors. 5 mg of nuclear lysate was used for immunoprecipitation using a mouse anti-p16^INK4a ^antibody or a matched mouse IgG. Protein antibody complexes were purified using protein-A-agarose (Santa Cruz, Santa Cruz, CA, USA). Immunoblotting was performed as described above, endogenous BRG1 was detected with a mouse anti-BRG1 antibody.

### Immunocytostaining

SW-13 cells were seeded at 10^5 ^cells on cover slips into 6-well plates and transfected 24 hours post seeding with 1 μg pCMV-MYC5b-p16 and 1.5 μg pcDNA3-BRG1-FLAG. Cells were fixed with methanol:acetone (1:1) for one minute, washed with PBS and probed with mouse anti-FLAG and rabbit anti-MYC antibodies and secondary Alexa Fluor 594 nm goat-anti-mouse and Alexa Fluor 488 nm goat anti-anti-rabbit antibodies (Invitrogen, Carlsbad, CA, USA). Images were taken with a BX-51 microscope and a SPOT camera and a FV1000 confocal microscope (Olympus, Center Valley, PA, USA).

WMM1175_p16^INK4a ^cells silenced for BRG1 or expressing a control silencing molecule were seeded after 1, 3, 5 days induction with 4 mM IPTG at 4 × 10^4 ^cells on cover slips and fixed 8 hours later with 2% formaldehyde, 0.2% glutaraldehyde, 7 mM Na_2_HPO_4_, 1.5 mM KH_2_PO_4_, 140 mM NaCl, and 2.6 mM KCl and stained for SA-β-galactosidase, DAPI, Ki67, p16^INK4a ^and BRG1. Images were taken with a BX-51 microscope and a SPOT camera (Olympus, Center Valley, PA, USA).

### Western blotting

50 μg total cell lysate or nuclear lysate was separated on 15% SDS-PAGE gels or 5–15% gradient SDS-page gels, transferred to PVDF membranes (Millipore, Billerica, MA, USA) and probed for β-actin p16^INK4a^, BRG1, BRM, pRb, CDK6, phoshorylated pRb and p16^INK4a^.

### Cell proliferation assay

WMM1175_p16^INK4a ^cells silenced for BRG1 or expressing a control silencing molecule were seeded at 10^3 ^cells per well in 96 well plates. For each day one plate was assayed for MTS levels using a CellTitre 96 Aqueous One Solution Proliferation assay (Promega, Madison, Wisconsin, USA) according to the manufacturer's protocol using a Victor^2 ^1420 Multilable counter (Perkin Elmer).

### Cell cycle distribution

10^5 ^cells were seeded per well into 6-well plates and 24 hours later transfected with 1 μg pCMV-MYC5b-p16 and/or 1.5 μg pcDNA3-BRG1-FLAG or 2.5 μg pCMV-MYC5b vector plus 250 ng pEGFPspectrin. Total transfected DNA was adjusted to 2.75 μg with pCMV-MYC5b vector. Cells were harvested 48 hours post transfection and fixed in 4°C 70% ethanol for at least 1 hour and stained with 50 μg/ml propidium iodide and 50 μg/ml RNasesA in PBS and analyzed using a FACScalibur and ModFit software (Becton Dickinson, Franklin Lakes, NJ, USA). Percent S-phase change was calculated (percent S-phase vector control – percent S-phase sample) × 100/percent S-phase vector control.

WMM1175_wtp16 cells expressing a siRNA targeting BRG1 or a control siRNA molecule targeting luciferase were induced for 1, 3 or 5 days with 4 mM IPTG or mock treated. For each time point the cell cycle distribution was determined as described above.

### Immunohistochemistry

Paraffin-embedded formaldehyde fixed primary (Breslow depth of invasion > 2 mm) or metastatic melanomas were cut at 4 mm onto Superfrost Plus slides and dried at 60°C for 1 hour. Sections were rehydrated through histolene and ethanol, heated in antigen retrieval buffer (Dako, Glostrup, Denmark) overnight at 70°C. Slides were placed in 3% hydrogen peroxide for 10 min then blocked for 1 hour with 50% normal goat serum (Serum Australis, Tamworth, NSW, Australia) diluted in 1% Tween 20/tris buffered saline (TTBS). Samples were incubated with primary antibodies for 1 hour at dilutions indicated. For p16^INK4a ^and BRG1 slides were incubated for 30 minutes with biotinylated goat anti-rabbit (Dako, Glostrup, Denmark) diluted 1:400 in TBT (1%BSA in TTBS) and finally for 30 minutes with biotinylated-HRP/streptavidin (Invitrogen, Carlsbad, CA, USA) diluted in TBT. Antibodies were detected using 3,3'-diaminobenzidine tetrachloride (DAB; Invitrogen, Carlsbad, CA, USA), counter stained with Mayers Haemotoxylin (Sigma, Castle Hill, NSW, Australia), dehydrated and mounted using Normount (Fronine, Riverstone, NSW, Australia). For BRM, slides were incubated for 1 hour with Alexa Fluor goat anti-rabbit 594 nm (Invitrogen, Carlsbad, CA, USA) diluted 1:1000 with DAPI (Sigma, Castle Hill, NSW, Australia) diluted 1:2000 in TBT. Slides were washed then mounted using 3% n-propylgallate/50% glycerol. Primary antibodies used were mouse anti-p16 (1:200), rabbit anti-BRG1 (1:100) and rabbit anti-BRM1 (1:400). Sections were scored for staining intensity as 0 (equal to control), 1 (very weak positive)), 2 (positive) and 3 (strong positive) and the proportion of tumor tissue with positive staining as 0 (none), 1 (< 10%), 2 (< 50%) and 3 (> 50%). Tumors were considered to have detectable positive staining when the (intensity score) × (proportion staining score) was > 1. Only tumor samples with enough tissue for staining of at least two of the proteins were included in the study. Appropriate negative and positive controls were used with each batch of immunostaining. This study is covered by the Sydney South West Area Health Service Ethics Review Committee (RPAH Zone) Protocol No. X08-0155 & HREC Ref. 08/RPAH/262 – "Histological and Immunohistological Analysis of Melanocytic Tumours".

## Competing interests

The authors declare that they have no competing interests.

## Authors' contributions

TB conceived and designed the project, carried out the initial Y2H screen, participated in and supervised most experimental work and drafted the manuscript. SH participated with endogenous IPs and carried out most of the senescence work. MD isolated and identified BRG1 from Y2H candidate clones and confirmed BRG1-p16INK4a interaction in human cells. LS participated in the design of the study and carried out the immunohistochemistry. MF carried out confocal microscopy. ED contributed her expertise in Y2H work. RS was critically involved in the immunohistochemistry analysis. DN contributed resources and was involved in the design of the study. GM was involved in the design of the study and the analysis. RK was involved in the design of the study and the analysis. HR participated in the senescence work and was critically involved in design and coordination of the study and the analysis and helped to draft the manuscript. All authors revised the manuscript and approved the final version.
